# Increased cytoplasmic and nuclear S100A6 expression is associated with improved prognosis in ovarian cancer

**DOI:** 10.1186/s12885-026-15631-0

**Published:** 2026-02-11

**Authors:** Aruba Farooq, Evren M. Akyuz, Brandon WQ Cheah, Suha Deen, Stewart G. Martin, Mattéa J. Finelli, Sarah Storr, Alan McIntyre

**Affiliations:** 1 Hypoxia and Acidosis Research Group, Centre for Cancer Sciences, School of Medicine, University of Nottingham Biodiscovery Institute, Nottingham, UK; 2 Department of Pathology, Queen’s Medical Centre, Nottingham University Hospitals NHS Trust, Nottingham, UK; 3 Nottingham Breast Cancer Research Centre, School of Medicine, University of Nottingham Biodiscovery Institute, Nottingham, UK; 4 School of Medicine, University of Nottingham Biodiscovery Institute, Nottingham, UK

**Keywords:** S100A6, Ovarian cancer, Survival

## Abstract

**Background:**

Ovarian cancer is one of the most common gynaecological cancers affecting more than 300,000 women worldwide each year. S100 calcium-binding protein A6 (S100A6) is a member of the S100 family of calcium-binding proteins. Upon activation by calcium (Ca^2+^) signalling, S100A6 regulates numerous cellular processes including cell proliferation and metastasis. The role of S100A6 is well established across multiple tumour types where increased S100A6 expression contributes to tumourigenesis and worse patient outcome. However, the role of S100A6 in ovarian cancer is not well established.

**Methods:**

The impact of cytoplasmic and nuclear S100A6 expression on overall survival and clinicopathological criteria was investigated in 462 ovarian tumours by immunohistochemistry. Additionally, *S100A6* expression in an ovarian cancer cohort from The Cancer Genome Atlas (TCGA) PanCancer dataset (*n* = 299) was evaluated and associations with overall survival and progression-free survival were identified.

**Results:**

Immunohistochemical staining revealed that high cytoplasmic expression of S100A6 was significantly associated with better overall survival (*p* = 0.014). Additionally, high nuclear expression was significantly associated with better overall survival (*p* = 0.036). In contrast, analysis of mRNA *S100A6* suggests no significant association of mRNA levels for overall survival (*p* = 0.903) and progression free survival (*p* = 0.278).

**Conclusions:**

Our data provides novel insights regarding the clinical implications of S100A6 expression in ovarian cancer, providing strong rationale for functional investigations of S100A6 in ovarian cancer.

**Supplementary Information:**

The online version contains supplementary material available at 10.1186/s12885-026-15631-0.

## Introduction

### Ovarian cancer

Cancer remains a global health concern with incidence and mortality increasing [1]. Ovarian cancer is a common gynaecological cancer affecting more than 300,000 women each year worldwide [1, 2]. In the UK, ovarian cancer is the sixth most common cancer in women and the fifth most common cause of cancer-related death [1]. The most common form of ovarian cancer is epithelial ovarian cancer, representing approximately 90% of cases; other forms include germ cell tumours and stromal tumours [3]. Epithelial ovarian cancer largely consists of five histological subtypes: high-grade serous carcinoma (HGSC), low-grade serous carcinoma (LGSC), endometrioid carcinoma, mucinous carcinoma, and clear-cell carcinoma [4]. Histological subtypes are differentiated by their morphology, mutational profile, and response to therapy [5]. Currently, standard treatment of ovarian cancer consists of surgery, and platinum-based adjuvant chemotherapy [6]. Common driver genetic alterations in ovarian cancer include tumour protein 53 (TP53), breast cancer gene 1/2 (BRCA1/2), phosphatidylinositol-4,5-bisphosphate 3-kinase catalytic subunit alpha (PIK3CA), and Kirsten rat sarcoma viral oncogene homolog (KRAS) [7, 8].

### S100A6

S100 calcium-binding protein A6 (S100A6), also referred to as calcyclin, is a member of the S100 family of proteins that includes at least 20 members [9]. The S100 proteins are calcium-binding and changes in their structure and activation are dependent upon Ca^2+^ signalling^10^. First purified from Ehrlich ascites carcinoma cells, S100A6, located on chromosome 1, encodes a low-molecular weight protein which binds to calcium via its EF-hand motifs [11, 12]. It is localised to the cytoplasm however it is also found in the nucleus, and upon activation by Ca^2+^ it can associate with the nuclear and cytoplasmic membrane [13–15]. S100A6 is expressed across multiple cell types including neurons [16], pancreatic cells [17], fibroblasts, and epithelial cells [18]. It interacts with multiple different targets found in the cytoplasm, nucleus, cell membrane, and extracellular matrix, through its exposed hydrophobic region following Ca^2+^ activation [13]. Through its interaction with the p53 protein, encoded by the TP53 gene, and other members of the p53 transcription factor family including p63 and p73, S100A6 is implicated in regulating cell proliferation, differentiation, and cell death [19, 20]. Furthermore, interactions with cytoskeletal proteins, such as actin and tropomyosin, highlights its role in cytoskeletal regulation and cell motility [15]. Other binding partners include calcyclin-binding protein/Siah-1-interacting protein (CacyBP-SIP), indicating a role in cell proliferation and differentiation, and receptor for advanced glycation end products (RAGE), indicating a role in the cellular stress response by inducing apoptosis through activation of c-Jun N-terminal kinase (JNK) via reactive oxygen species (ROS) [21, 22]. Additionally, studies have noted a relationship between S100A6 and β-catenin activation, an activator of the Wnt signalling pathway [23, 24]. Its numerous functions in physiology implicates S100A6 in multiple cancer types due to aberrant expression, as well as other diseases such as cardiovascular disease, endometriosis, and Alzheimer’s disease [25]. As a result, S100A6 is involved in numerous cellular processes.

Increased levels of S100A6 have been investigated and linked with tumourigenesis and altered survival outcome in numerous cancer types [10]. For example, in pancreatic cancer high levels of S100A6 are associated with adverse survival, through promotion of epithelial-to-mesenchymal transition and cell motility, with investigators also postulating a role of S100A6 as a biomarker in pancreatic cancer [24, 26–28]. In hepatocellular and cervical cancer, elevated levels of S100A6 are associated with activation of the phosphoinositide 3-kinase/protein kinase B (PI3K/Akt) pathway, promoting cancer cell proliferation and migration [29, 30]. In gastric cancer, upregulation of S100A6 is associated with increased tumour cell proliferation and worse patient outcome [31, 32].

Of the S100 proteins, current evidence presents a unique perspective of the role of S100A6 in ovarian cancer progression and patient survival. Bai et al. found that higher S100A6 protein expression was associated with improved overall survival (OS) in ovarian cancer, however survival analysis of different stages indicates that the effect of increased S100A6 expression differs across early and late-stage disease [33]. Previous murine studies have shown that serum levels of S100A6 are increased in advanced stage ovarian cancer, suggesting the potential for S100A6 as a biomarker, in combination with other biomarkers, for ovarian cancer detection, however there is little data to support its utility in this respect [34]. This study aims to increase our understanding of S100A6 and its associations with patient survival across different groups of ovarian cancer patients, using bioinformatics analysis of publicly available datasets and immunohistochemical analysis of S100A6 protein expression in a cohort of 462 ovarian tumours.

## Materials and methods

### Western blotting

Specificity of the S100A6 antibody (10245-1-AP, Proteintech) was initially determined via Western Blotting, to assess suitability for subsequent IHC based studies. OVCA433 cell line is representative of serous epithelial ovarian cancer which constitute the largest proportion of epithelial ovarian cancers. SKOV-3 cell line is representative of non-serous ovarian cancer and was used as an additional cell line for validation. OVCA433 was kindly gifted by Professor Lindy Durrant (University of Nottingham, UK) and SKOV-3 was purchased from the American Type Culture Collection (ATCC). OVCA433 and SKOV-3 were cultured in Dulbecco’s Modified Eagle Medium (DMEM) (10566016, Thermo Fisher Scientific) supplemented with 10% fetal bovine serum (FBS) (F9665, Sigma-Aldrich). All cells were maintained at 37 °C and 5% CO_2_. Cells were short tandem repeat profiled and were routinely tested for mycoplasma. Lysates from OVCA433 and SKOV-3 ovarian cancer cells were prepared using cell lysis buffer, consisting of radioimmunoprecipitation assay (RIPA) buffer (9806, Cell signalling), and protease inhibitor (P8340, Sigma-Aldrich). A Bradford assay using the protein assay dye reagent concentrate (5000006, Bio-Rad) was conducted for protein quantification, readings were taken using the Infinite F50 (Tecan) and quantified using Magellan software. Protein extracts were prepared with Lamelli buffer (1610747, Bio-Rad) with 10% beta-mercaptoethanol (M3148, Sigma-Aldrich) and denatured at 95 °C for 5 min. 15% Polyacrylamide gels were used for gel electrophoresis. Equal amounts of protein were loaded along with the Precision Plus Protein Dual Colour Standards ladder (1610374, Bio-Rad). Proteins were transferred to a 0.2 μm nitrocellulose membrane (1704270, Bio-Rad), blocked with 5% skimmed milk (70166, Sigma-Aldrich) in tris-buffered saline (TBS) with 0.1% Tween-20 (TBS-T) (P7949, Sigma-Aldrich) for 60 min, and incubated with S100A6 (1:500) and β-actin (1:5000) (SC-47778, Santa Cruz Technology) antibodies overnight at 4 °C. The next day membranes were washed with TBS-T and then incubated with secondary antibodies (1:5000) anti-mouse IRDye^®^ 680RD (926–68070, LI-COR) and anti-rabbit IRDye^®^ 800RD (926–32211, LI-COR) diluted in 5% milk for 60 min at room temperature. Membranes were imaged using the Odyssey^®^ XF Imaging System (LI-COR) and quantified using Image Studio™ Lite (V5.5.4)(Supplementary Fig. 2).

### Patient cohorts

The cohort of patients, used for IHC studies, were treated between 1991 and 2011 at Nottingham University Hospitals. The tissue microarray (TMA) used in this study has been described previously [35]. Overall survival was defined as the time between the start of treatment and date of death or last follow up date. Progression-free survival was defined as the time between the start of treatment and recurrence of last follow-up date. Age was categorised based on the median age of the patient cohort. Data on chemotherapy resistance were recorded according to the Gynaecological Oncology Group (COG) as refractory (not responding to chemotherapy), resistant (an initial response to chemotherapy with recurrence within 6 months) or sensitive (either no recurrence, or recurrence after 6 months). Suboptimal debulking was classified as residual disease of > 2 cm. Clinicopathological information available includes patient age, FIGO stage, tumour grade, histological subtype, and residual disease; shown in Table 1. Medium follow up was 100 months which was determined using the reverse Kaplan-Meier method. Ethical approval was obtained from Derbyshire Ethics Committee (07/H0401/156). This study is reported in accordance with REMARK criteria [36].

### TCGA ovarian cancer cohort

The TCGA patient cohort has been described previously [37]. Patient samples were collected at contributing centres following informed consent at their local institutional review boards. Data was downloaded from https://www.cbioportal.org/ and analysis was carried out on mRNA expression z-scores relative to all diploid samples (log RNASeq V2 RSEM).

### Immunohistochemistry

Immunohistochemical (IHC) staining was performed using the Novolink Polymer Detection Systems kit (RE7140-CE, Leica Biosystems) on TMAs comprising of single 0.6 mm cores from 575 ovarian tumours; staining was performed following the manufacturer’s instructions. Slides were deparaffinised with xylene and then rehydrated in ethanol then water. Heat induced epitope retrieval with sodium citrate buffer at pH 6.0 was used to retrieve antigens. Slides were incubated with the Novolink Peroxidase Block, washed with TBS-T, and then incubated with the Novolink Protein Block. Slides were stained with S100A6 diluted at 1:250 in BOND primary antibody diluent (AR9352, Leica Biosystems) for 60 minutes at room temperature. Following TBS-T washes, slides were then incubated with the Novolink Polymer solution for 30 minutes. Staining was identified using the chromogenic substance 3,3’ diaminobenzidine, counterstained with haematoxylin. Slides were imaged using the NanoZoomer Digital Pathology Scanner (Hamamatsu Photonics) and analysed with NDP.view2 (Hamamatsu Photonics). Cytoplasmic staining was assessed via a semi-quantitative immunohistochemical H score, with staining intensity categorised as none (0), weak (1), medium (2), or strong (3) multiplied by the percentage area for each score, ranging from 0 to 300. Nuclear staining was assessed via a semi-quantitative method, whereby the percentage of cores that demonstrated any nuclear staining were scored positive. More than 30% of cores from the cohort were independently scored by a second assessor, with both assessors blinded to each other’s scores, clinicopathological, and survival data.

### Statistical analysis

Statistical analysis for both patient cohorts was performed using IBM SPSS statistics version 29.0.1.0. Cut points for high and low expression for survival were identified using X-Tile [38]. Survival curves were plotted using the Kaplan-Meier method and significance determined using the log-rank test. Pearson’s *χ*^2^ test of association was used to assess the association of protein expression and clinicopathological variables. Multivariate survival analysis was performed with Cox proportional hazards regression model. Spearman’s rank correlation coefficient was performed to assess cytoplasmic and nuclear correlation. Results were deemed statistically significant at *p* < 0.05. Intraclass correlation coefficient (ICC) was used to determine correlation of cytoplasmic and nuclear staining assessments between first and second scorers, with the observed ICC > 0.8 indicating a high level of agreement.

### Additional bioinformatics

Online databases were utilised to assess S100A6 expression in ovarian cancer. Gene expression data of normal pancreatic samples from Genotype-tissue Expression (GTEx) and pancreatic tumour samples from the TCGA were obtained from OncoDB and analysed with GraphPad Prism 10.6.1 [39]. S100A6 normal vs. tumour comparison was performed on TNMplot [40]. Kaplan-Meier plotter was used to plot overall survival data [41]. Further analyses of S100A6 on survival and expression across different subtypes was performed using Gene Expression database of Normal and Tumour tissues (GENT2) [42]. Data is considered significant when *p* < 0.05.

## Results

### Cytoplasmic and nuclear S100A6 protein staining location and frequency

**Fig. 1:**
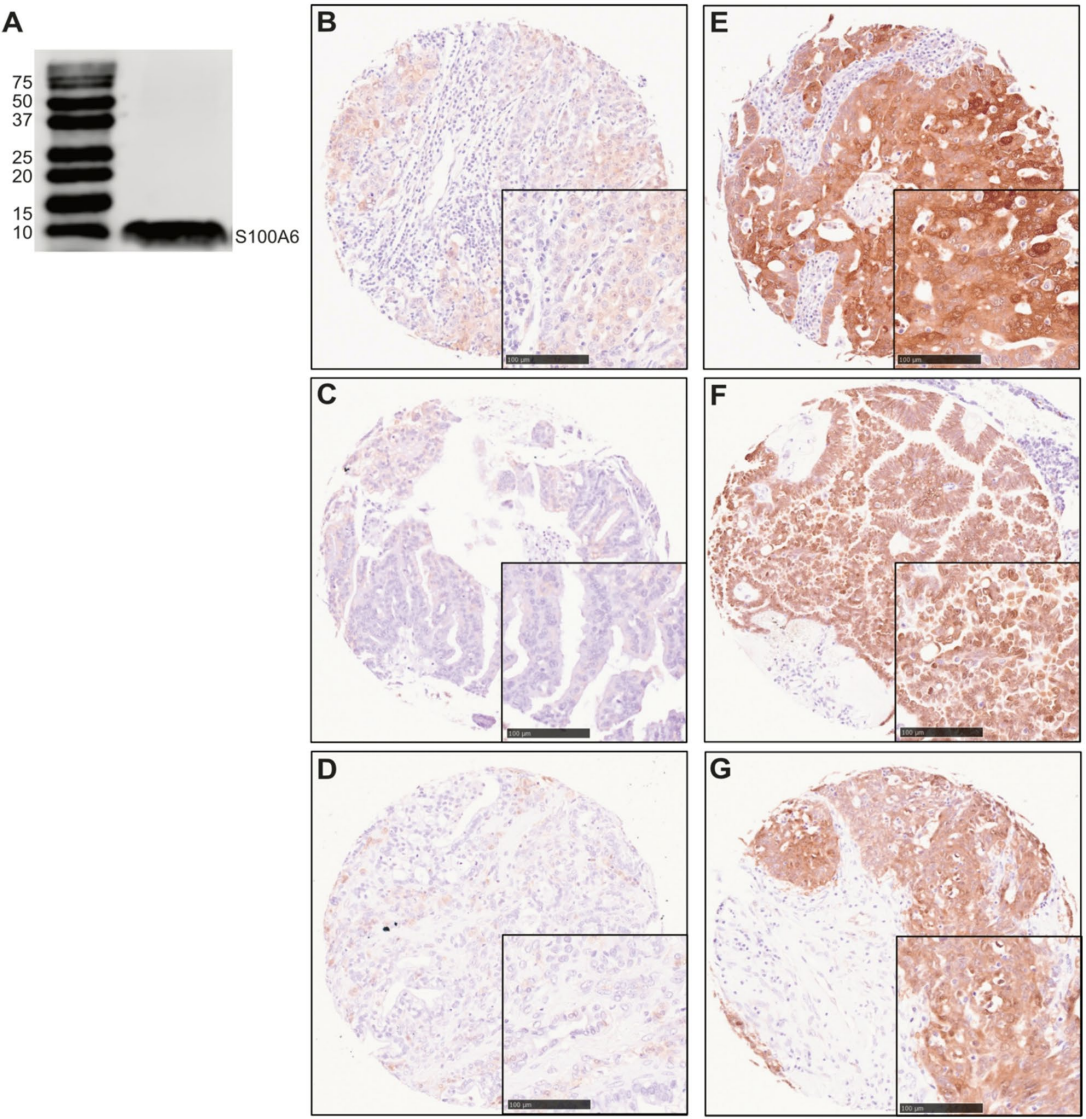
(**A**) Western blot of S100A6 in OVCA433 ovarian cancer cells, with an expected single band of 10 kDa. Full-length blots with an additional cell line (SKOV-3) are presented in Supplementary Fig. 2. Representative images of IHC staining for low (**B-D**) and high (**E-G**) cytoplasmic and nuclear expression of S100A6. 10X images with a 20X index box. Scale bar 100 μm

S100A6 expression significantly increases in ovarian cancer patients compared to normal ovarian tissue when analysed from the OncoDB (*p* < 000.1) and TNMplot (*p* < 0.0001) online databases (Supplementary Fig. 1A-B) [39, 40]. Overall survival analysis from Kaplan-Meier plotter highlights no significant difference in survival (*p* = 0.51), however there is significance when analysed on GENT2 (*p* < 0.001) (Supplementary Fig. 1C-D) [41, 42]. Subtype separation indicate higher S100A6 expression in serous carcinomas (Supplementary Fig. 1E-F). In order to investigate this further, we assessed S100A6 expression in an ovarian cancer TMA, which provides robust information regarding associations between S100A6 and clinical outcome. To ensure that our chosen antibody is suitable for our study, its specificity was tested using Western blotting. Figure 1A shows the antibody is binding to the expected band at 10 kDa. The ovarian cancer TMA was subsequently stained with S100A6, and 462 of the 575 cases were available for assessment. H-scores for cytoplasmic staining ranged from 0 to 290, with a median H-score of 40 observed. A cut point of 100 was identified using X-Tile to distinguish high and low expression. Cytoplasmic and nuclear staining of S100A6 were identified, with variation in staining intensity observed between patient tumours (Fig. 1B-G). Cytoplasmic and nuclear S100A6 correlation was assessed using Spearman’s rank correlation coefficient analysis. Cytoplasmic and nuclear S100A6 expression significantly correlate in our cohort (ρ = 0.425, *n* = 462, *p* < 0.001).

### Relationship of S100A6 protein expression with clinicopathological criteria

We conducted more detailed analyses of the association of cytoplasmic and nuclear S100A6 expression with clinicopathological criteria by performing Pearson’s chi-squared tests (Table 1). Higher cytoplasmic S100A6 expression was significantly associated with patient age (*χ*^2^ = 7.619, d.f.=1, *p* = 0.006), higher International Federation of Gynaecology and Obstetrics (FIGO) stage (*χ*^2^ = 13.072, d.f.=3, *p* = 0.004), grade 3 tumours (*χ*^2^ = 37.984, d.f.=2, *p* < 0.001), presence of residual disease (*χ*^2^ = 6.896, d.f.=2, *p* = 0.032), and tumour histology (*χ*^2^ = 65.349, d.f.=8, *p* < 0.001). Nuclear S100A6 expression was associated with patient age (*χ*^2^ = 11.123, d.f.=1, *p* < 0.001), higher FIGO stage (*χ*^2^ = 8.186, d.f.=3, *p* = 0.042), grade 3 tumours (*χ*^2^ = 17.364, d.f.=2, *p* < 0.001), and tumour histology (*χ*^2^ = 74.089, d.f.=8, *p* < 0.001).

**Table 1:** Relationship of cytoplasmic and nuclear S100A6 expression with clinicopathological variables determined using immunohistochemistry

	Cytoplasmic S100A6 expression			Nuclear S100A6 expression		
	Low	High	*p* value	Low	High	*p* value
Patient Age						
61 years and below	130 (27.4%)	98 (20.6%)	**0.006**	162 (34.1%)	66 (13.9%)	**< 0.001**
Older than 61	171 (36.0%)	76 (16.0%)		207 (43.6%)	40 (8.4%)	
Figo stage						
1	90 (19.1%)	78 (16.6%)	**0.004**	119 (25.3%)	49 (10.4%)	**0.042**
2	42 (8.9%)	13 (2.8%)		46 (9.8%)	9 (1.9%)	
3	143 (30.4%)	69 (14.6%)		174 (36.9%)	38 (8.1%)	
4	25 (5.3%)	11 (2.3%)		27 (5.7%)	9 (1.9%)	
Tumour grade						
1	16 (3.4%)	23 (4.8%)	**< 0.001**	28 (5.9%)	11 (2.3%)	**< 0.001**
2	30 (6.3%)	47 (9.9%)		47 (9.9%)	30 (6.3%)	
3	256 (53.9%)	103 (21.7%)		295 (62.1%)	64 (13.5%)	
Residual disease						
No residual tumour	153 (36.4%)	108 (25.7%)	**0.032**	198 (47.1%)	63 (15.0%)	0.717
Residual tumour < 2 cm	38 (9.0%)	12 (2.9%)		40 (9.5%)	10 (2.4%)	
Residual tumour > 2 cm	74 (17.6%)	35 (8.3%)		86 (20.5%)	23 (5.5%)	
Histology						
High-grade serous carcinoma	191 (40.1%)	86 (18.1%)	**< 0.001**	228 (47.9%)	49 (10.4%)	**< 0.001**
Mucinous	21 (4.4%)	27 (5.7%)		36 (7.6%)	12 (2.5%)	
Endometrioid	35 (7.4%)	19 (4.0%)		50 (10.5%)	4 (0.8%)	
Clear cell carcinoma	40 (8.4%)	6 (1.3%)		33 (6.9%)	13 (2.7%)	
Mixed	1 (0.2%)	2 (0.4%)		2 (0.4%)	1 (0.2%)	
Other epithelial	9 (1.9%)	2 (0.4%)		11 (2.3%)	0 (0.0%)	
Low-grade serous carcinoma	3 (0.6%)	21 (4.4%)		9 (1.9%)	15 (3.2%)	
Borderline serous carcinoma	2 (0.4%)	10 (2.1%)		1 (0.2%)	11 (2.3%)	
Borderline mucinous carcinoma	0 (0.0%)	1 (0.2%)		0 (0.0%)	1 (0.2%)	

The *p* values are resultant from Pearson’s *χ*^2^ test of association; significant values are highlighted in bold

### Association of S100A6 protein expression with overall survival

**Fig. 2:**
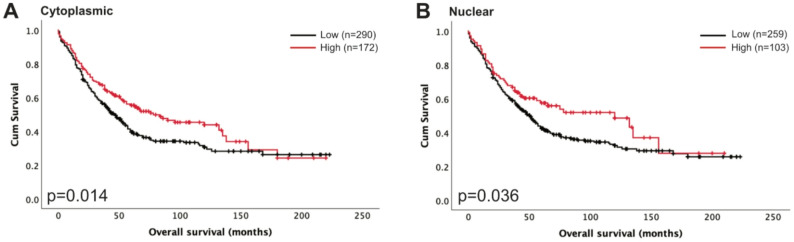
Kaplan-Meier analysis of ovarian cancer overall survival with low (black) and high (red) S100A6 expression in the cytoplasm (**A**) and nucleus (**B**). Significance was determined using the log-rank test

Analysis of associations with clinical data identified that high cytoplasmic S100A6 expression was significantly associated with better OS (*p* = 0.014, *n* = 462, Fig. 2A). However, multivariate Cox regression analysis reveals no significance (*p* = 0.225, hazard ratio = 0.835, 95% confidence interval = 0.623–1.118) when including FIGO stage, residual disease, tumour grade, and histology, which all had individual Kaplan-Meier log rank statistics *p* < 0.05. Additionally, nuclear S100A6 expression was associated with better OS (*p* = 0.036, *n* = 462, Fig. 2B). Similarly, multivariate Cox regression analysis reveals no significance (*p* = 0.578, hazard ratio = 0.905, 95% confidence interval = 0.636–1.287) when including FIGO stage, residual disease, tumour grade, and tumour histology, which all had individual Kaplan-Meier log rank statistics *p* < 0.05. We were unable to assess progression-free survival (PFS) as there were not enough events.

### Association of S100A6 expression with histological subtype

**Fig. 3:**
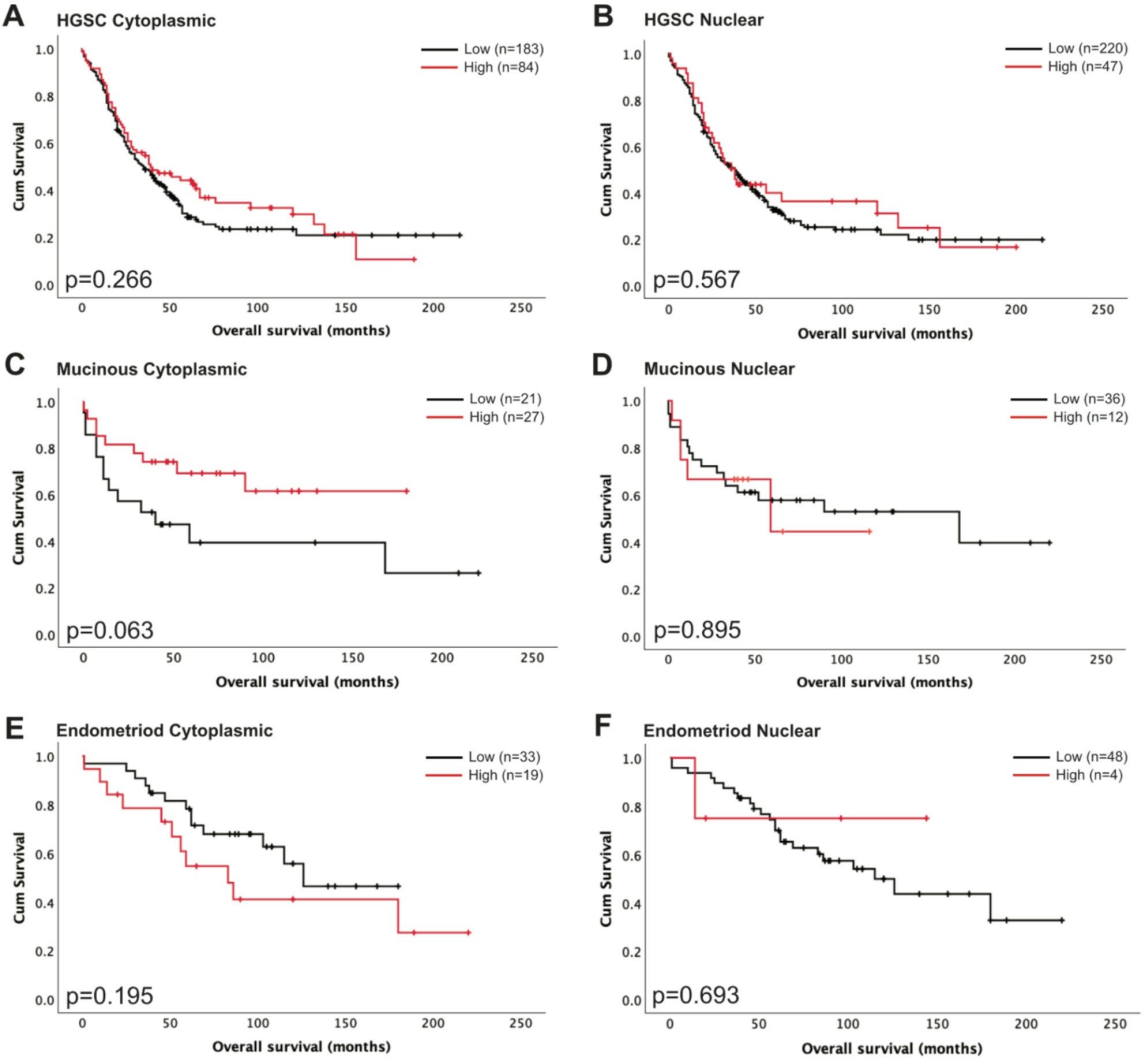
Kaplan-Meier analysis of ovarian cancer overall survival with low (black) and high (red) S100A6 expression in the cytoplasm (**A**, **C**, **E**) and nucleus (**B**, **D**, **F**) in high-grade serous carcinoma (HGSC) (**A**-**B**), mucinous carcinoma (**C**-**D**), and endometrioid carcinoma (**E**-**F**). Significance was determined using the log-rank test

Cytoplasmic and nuclear S100A6 expression levels were further assessed in different histological subtypes of ovarian cancer. In HGSC cases, S100A6 expression was not significantly associated with worse patient survival when expressed in the cytoplasm (*p* = 0.266, *n* = 267, Fig. 3A) or the nucleus (*p* = 0.567, *n* = 267, Fig. 3B). In mucinous carcinoma cases, S100A6 expression was not significantly associated with worse patient survival in the cytoplasm (*p* = 0.063, *n* = 48, Fig. 3C) or the nucleus (*p* = 0.895, *n* = 48, Fig. 3D). In endometrioid carcinoma cases, S100A6 expression was not significantly associated with worse patient survival in the cytoplasm (*p* = 0.195, *n* = 52, Fig. 3E) or the nucleus (*p* = 0.693, *n* = 52, Fig. 3F). Other histological subtypes were not assessed due to limited cases available.

### Transcriptomic analysis in the TCGA cohort

**Fig. 4:**
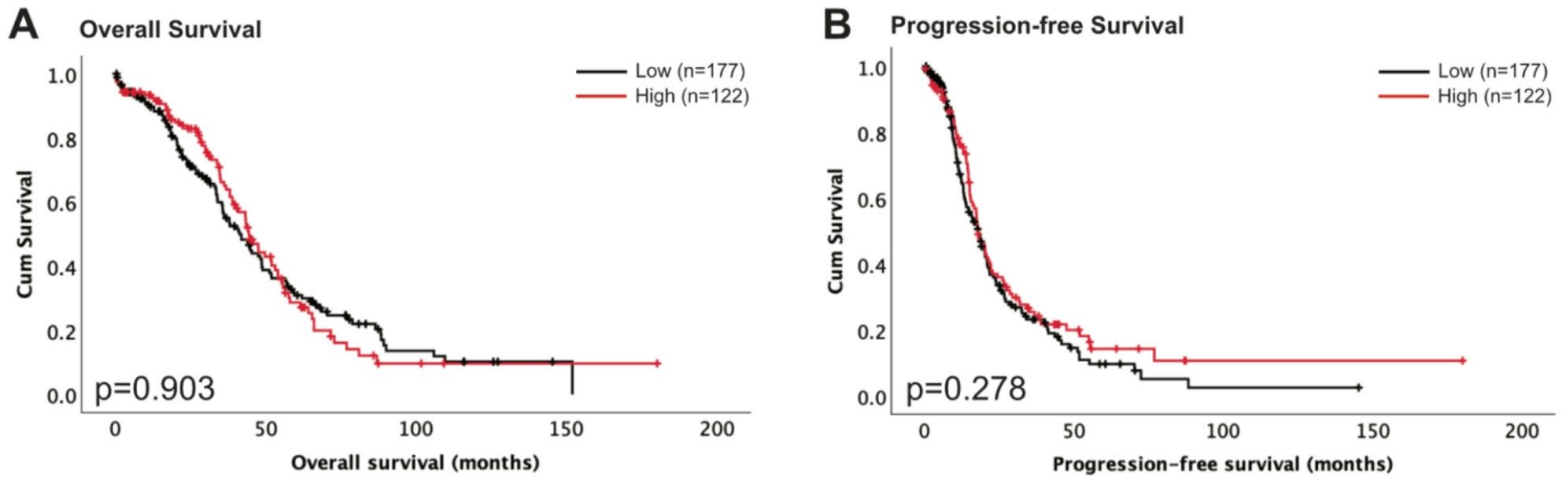
Kaplan-Meier analysis of overall survival (**A**) and progression free survival (**B**) with low (black) and high (red) mRNA expression of S100A6 in ovarian cancer. Significance was determined using the log-rank test

*S100A6* mRNA expression was assessed to explore its association with OS and progression-free survival (PFS) in ovarian cancer patients, from publicly available data from the TCGA patient cohort containing ovarian serous cystadenocarcinoma cases, to assess similarity to our cohort. 299 cases were available for assessment. *S100A6* expression ranged from − 1.23 to 23.06. A cutpoint of −0.12 was determined by X-tile. Higher expression of *S100A6* (*n* = 122) was not associated with worse OS (*p* = 0.903, *n* = 299, Fig. 4A) or worse PFS (*p* = 0.278, *n* = 300, Fig. 4B).

### Association of *TP53* mutation with *S100A6* expression

TP53 is frequently mutated in ovarian cancer and previous studies have characterised the relationship between S100A6 and p53 protein, identifying p53 as a key binding partner [19, 20]. Therefore, we investigated whether *TP53* mutation was associated with changes in *S100A6* expression. We stratified the TCGA ovarian cancer patient cohort into wildtype *TP53* or mutant *TP53* and assessed for association with categorised *S100A6* expression. Pearson’s chi-squared tests were performed to evaluate the relationship between *TP53* mutation and *S100A6* expression. *TP53* mutation was not associated with *S100A6* expression (*χ*^2^ = 0.417, d.f.=1, *p* = 0.723) (Supplementary Table 1).

## Discussion

S100A6 has been extensively investigated in a range of tumour types, showing associations of increased S100A6 expression with worse patient outcome through its contribution to tumourigenesis. Previous studies have described how S100A6 expression in ovarian cancer differs across early and late-stage disease in serous ovarian adenocarcinoma models [34]. Whilst others have alluded to improved survival outcome through dataset analyses on grouped cohorts of ovarian cancer patients [33]. This current study aimed to understand the impact of S100A6 on patient survival via immunohistochemical analysis using a large ovarian cancer TMA cohort.

S100A6 cytoplasmic and nuclear expression was investigated in the ovarian cancer cohort to assess associations with survival. Increased cytoplasmic and nuclear expression of S100A6 was associated with better survival outcome in ovarian cancer patients (*p* = 0.014 and *p* = 0.036, respectively). PFS data was not available for all cases therefore we were unable to assess the impact of high S100A6 expression on PFS. Cytoplasmic S100A6 was associated with patient age, higher tumour grade, higher FIGO stage, tumour histology and the presence of residual disease. Similarly, nuclear S100A6 was associated with patient age, higher tumour grade, higher FIGO stage, and tumour histology. We further divided our cohort into the different histological subtypes and investigated the impact of cytoplasmic and nuclear S100A6 expression. Of the five histological subtypes, we were only able to assess survival in HGSC, mucinous carcinoma, and endometrioid carcinoma due to limited cases available of the other histological subtypes. Our analysis showed that there was no significant association of high cytoplasmic or nuclear S100A6 expression with survival. Key signalling pathways and mutational profiles differ across each histological subtype [7]. Despite showing no significance, our analysis shows indications of differences that warrant further investigation using a larger dataset. Future studies should look to increasing sample size for more robust statistical analysis on OS and PFS, whilst also including further information regarding patient stage and grade, in order to provide robust analyses to determine the role of S100A6 in patient outcome.

*S100A6* mRNA expression was then assessed to determine whether there were associations with OS or PFS in the TCGA ovarian cancer cohort, of which increased *S100A6* expression did not correlate with worse survival outcome. We further explored the relationship between *TP53* mutation and *S100A6* expression. Correlation analysis of *TP53* mutation data from the TCGA cohort with *S100A6* expression revealed no associations. *TP53* is frequently mutated in ovarian cancer and remains common across all histological subtypes [43]. Mutant *TP53* has previously been described as a potential driver of ovarian cancer progression, which may explain why there is no correlation with S100A6 [44]. Previous studies have explored the correlation of protein levels of S100A6 and p53 [20]. Future investigation could include analyses of larger cohorts with p53 protein expression correlated to S100A6 expression to provide further insight into this relationship. The TCGA dataset provides an insight into *S100A6* expression on patient outcome in serous carcinoma only. Through utilising databases including Gene Expression Profiling Interactive Analysis for mRNA expression predictions using RNA sequencing data, and SurvExpress for survival analysis, Xu et al. performed comprehensive analysis on the S100 family in ovarian cancer [45]. Interestingly, they find that increased *S100A6* mRNA expression is correlated with better survival outcome, highlighting the importance of utilising larger datasets to assess correlation of protein expression with survival outcome [45].

Differences in S100A6 expression could correlate to treatment efficacy. In breast cancer, it has been shown that high expression of S100A6 is an indicator of pathologic complete response, through negatively regulating the oncoprotein mouse double minute 2 (MDM2), by inducing its degradation. Therefore, increased S100A6 expression sensitises breast cancer cells to chemotherapy through preventing chemoresistance [46]. In ovarian cancer treatment options include poly-ADP ribose polymerase (PARP) inhibitors and platinum-based chemotherapy [5]. Our findings show that high expression of S100A6 is associated with better survival outcome. Building upon the breast cancer study, further investigations could look into whether increased S100A6 expression sensitises ovarian cancer cells to chemotherapy, as this may provide some insight into how high S100A6 expression improves survival.

Activation of S100A6 is Ca^2+^ dependent, enabling its interactions with downstream targets [11, 12]. Increased calcium signalling has been implicated in cancer, with roles in cell proliferation, apoptosis regulation, and autophagy [47]. The potential of targeting calcium signalling in ovarian cancer has been reviewed previously [48]. Understanding the pattern of calcium signalling in ovarian cancer could potentially provide an insight into the effect of nuclear or cytoplasmic localisation on the downstream activation of S100A6. Therefore, further investigations are required to understand the functional role of S100A6, in addition to protein expression levels, and which proteins it is interacting with in ovarian cancer, to ultimately understand its association to improved survival. Binding partners of S100A6 have previously been identified, including CacyBP-SIP and RAGE, linking S100A6 to cell proliferation, differentiation, and apoptosis [21, 22]. Assessment of the relationship of these binding partners with S100A6 in ovarian cancer could provide better insight into which pathways are activated as a result of increased S100A6 expression. Following on from assessments by Bai et al. where increased S100A6 expression had opposing associations on OS across early and late-stage disease; future studies could be expanded to include S100A6 expression across different stages and grades of ovarian cancer, in addition to histological subtypes [33]. This could potentially include investigations of the functional role of S100A6 across human ovarian cancer cell lines that have been categorised into their histological subtypes.

## Conclusion

High cytoplasmic and nuclear S100A6 protein expression were associated with better survival in ovarian cancer patients. Assessment of survival across the different histological subtypes revealed no significant association. Future investigations into the functional role of S100A6 in ovarian cancer may provide clearer insight into the interacting partners and pathways involved that lead to better survival outcome. Additionally, a larger follow-up study with increased number of patients across different stages, grades, and histological subtypes of ovarian cancer could provide meaningful results to elucidate the role of S100A6 in ovarian cancer subtypes, and stages.

## Supplementary Information

Supplementary Material 1. Supplementary Figure 1: Analysis of S100A6 expression across different databases. A. normal vs tumour analysis of S100A6. Data obtained from OncoDB. T-test. ****p<0.0001. B. Normal vs tumour analysis of S100A6 performed on TNMplot. C. Kaplan-Meier analysis of S100A6 on ovarian cancer overall survival from Kaplan-Meier plotter. Impact of S100A6 on overall survival via Kaplan-Meier analysis on all ovarian cancer patients (D) and separated into subtypes (E) from GENT2. F. S100A6 expression across the different ovarian cancer subtypes from GENT2. Supplementary Figure 2: Uncropped Western blot image of S100A6 (10kDa) and β-Actin (42kDa) in OVCA433, N=3 (A) and SKOV-3, N=3 (B). Supplementary Table 1: Correlation analysis of TP53 and S100A6

## Data Availability

The TCGA mRNA expression data presented in this study are available for download (http://www.cbioportal.org), with immunohistochemistry data available on request.

## Abbreviations

S100A6: S100 calcium-binding protein A6
TCGA: The Cancer Genome Atlas
HGSC: High-grade serous carcinoma
LGSC: Low-grade serous carcinoma
TP53: Tumour protein 53
BRCA1/2: Breast cancer gene ½
PIK3CA: Phosphatidylinositol-4,5-bisphosphate 3-kinase catalytic subunit alpha
KRAS: Kirsten rat sarcoma viral oncogene homolog
CacyBP-SIP: Calcyclin-binding protein/Siah-1-interacting protein
RAGE: Receptor for advanced glycation end
JNK: c-Jun N-terminal kinase
ROS: Reactive oxygen species
PI3K/Akt: Phosphoinositide 3-kinase/protein kinase B
OS: Overall survival
TMA: Tissue microarray
FIGO: International Federation of Gynaecology and Obstetrics
PFS: Progression-free survival
MDM2: Mouse double minute 2
PARP: Poly-ADP ribose polymerase
