# Supplementary material for: Increased cytoplasmic and nuclear S100A6 expression is associated with improved prognosis in ovarian cancer
Source: BMC Cancer. 2026 Feb 11;26:368. doi: 10.1186/s12885-026-15631-0 (PMC12998080; doi:10.1186/s12885-026-15631-0)
Supplement: Supplementary file 1 — Supplementary Material 1. Supplementary Figure 1: Analysis of S100A6 expression across different databases. A. normal vs tumour analysis of S100A6. Data obtained from OncoDB. T-test. ****p<0.0001. B. Normal vs tumour analysis of S100A6 performed on TNMplot. C. Kaplan-Meier analysis of S100A6 on ovarian cancer overall survival from Kaplan-Meier plotter. Impact of S100A6 on overall survival via Kaplan-Meier analysis on all ovarian cancer patients (D) and separated into subtypes (E) from GENT2. F. S100A6 expression across the different ovarian cancer subtypes from GENT2. Supplementary Figure 2: Uncropped Western blot image of S100A6 (10kDa) and β-Actin (42kDa) in OVCA433, N=3 (A) and SKOV-3, N=3 (B). Supplementary Table 1: Correlation analysis of TP53 and S100A6 [file 12885_2026_15631_MOESM1_ESM.docx]

**Supplementary Data:**

**Supplementary Figure 1: Analysis of S100A6 expression across different databases. A. normal vs tumour analysis of S100A6. Data obtained from OncoDB. T-test. ****p<0.0001. B. Normal vs tumour analysis of S100A6 performed on TNMplot. C. Kaplan-Meier analysis of S100A6 on ovarian cancer overall survival from Kaplan-Meier plotter. Impact of S100A6 on overall survival via Kaplan-Meier analysis on all ovarian cancer patients (D) and separated into subtypes (E) from GENT2. F. S100A6 expression across the different ovarian cancer subtypes from GENT2.**

**
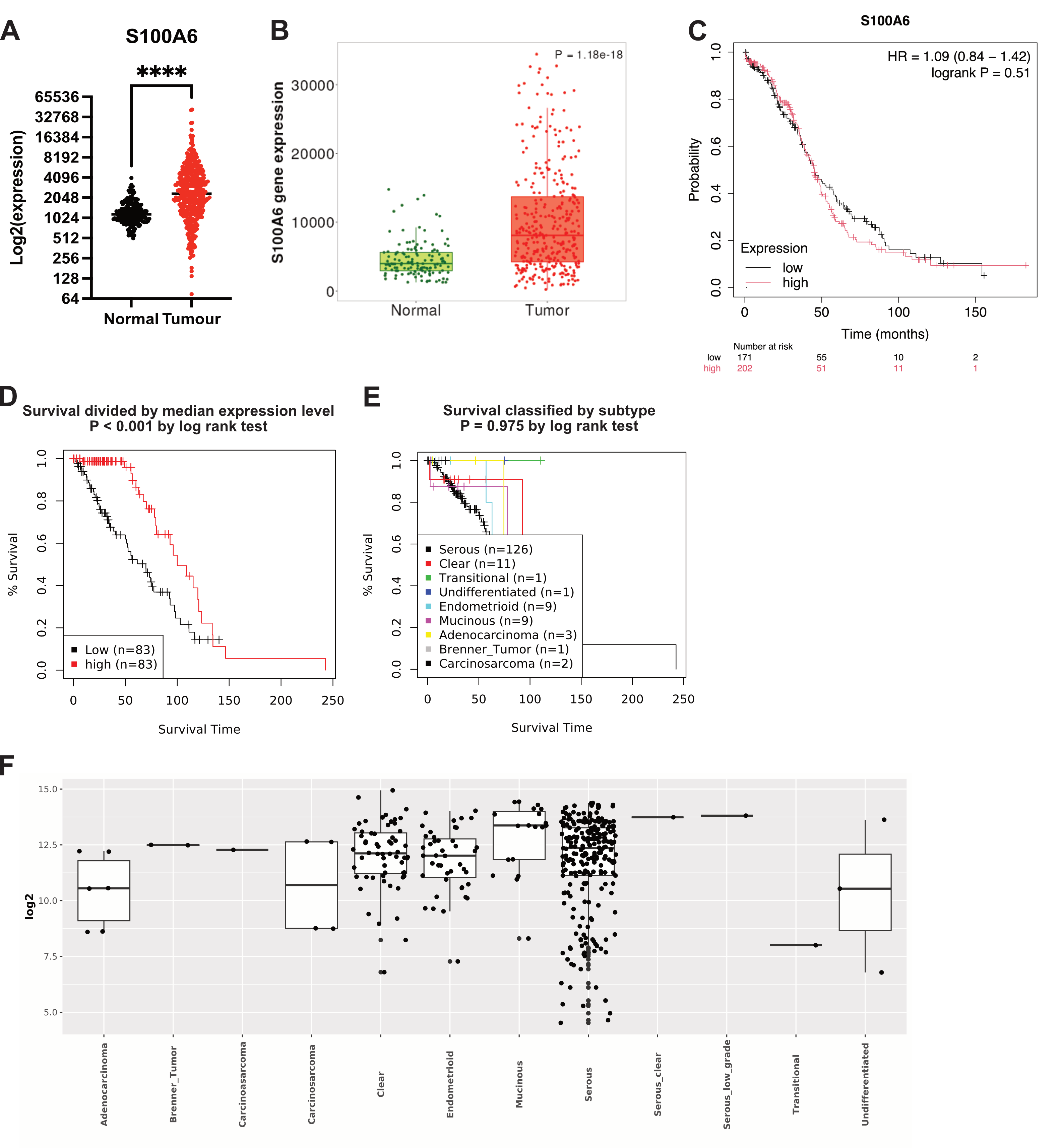
**

**Supplementary Figure 2: Uncropped Western blot image of S100A6 (10kDa) and β-Actin**

**(42kDa**) **in OVCA433, N=3 (A) and SKOV-3, N=3 (B).**

**
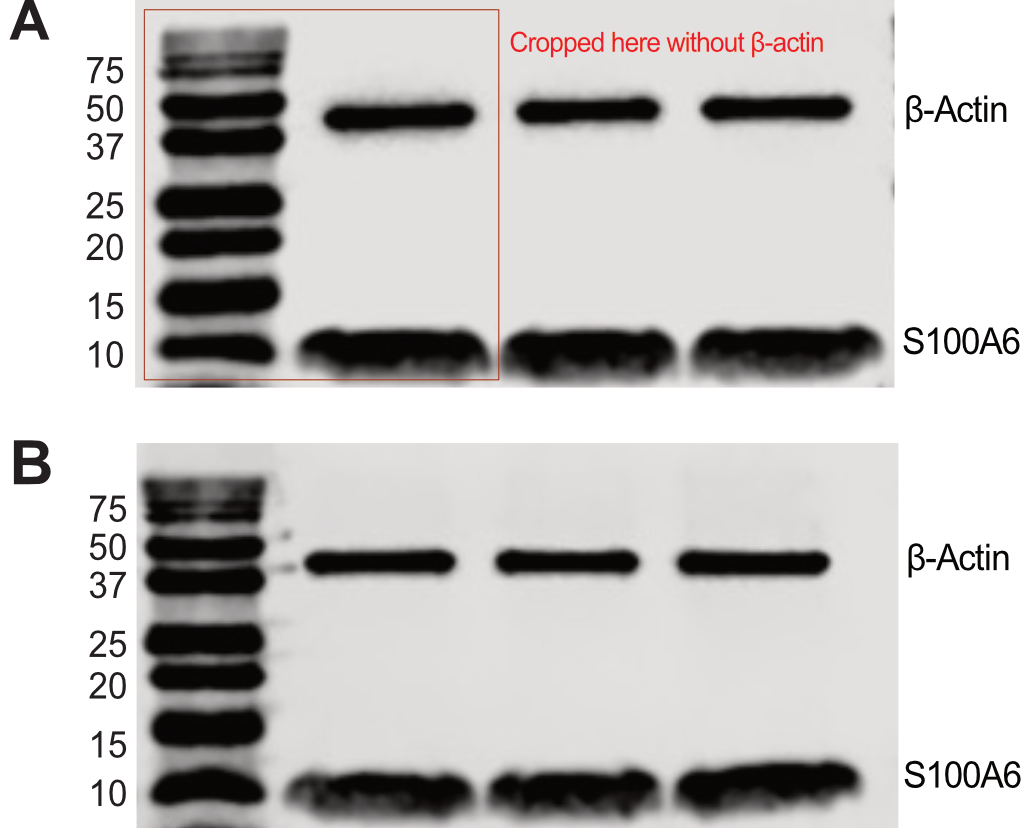
**

**Supplementary Table 1: Correlation analysis of *TP53* and *S100A6*.**

|  | **Value** | **df** | **Asymptotic Significance (2-sided)** | **Exact Sig. (2-sided)** | **Exact Sig. (1-sided)** |
| --- | --- | --- | --- | --- | --- |
| Pearson Chi-Square | 0.126 | 1 | 0.723 |  |  |
| Continuity Correction | 0.052 | 1 | 0.819 |  |  |
| Likelihood Ratio | 0.126 | 1 | 0.723 |  |  |
| Fisher’s Exact Test |  |  |  | 0.800 | 0.411 |
| Linear-by-Linear Association | 0.125 | 1 | 0.723 |  |  |
| N of Valid Cases | 291 |  |  |  |  |
